# Accurate Peak Detection for Optical Sensing with Reduced Sampling Rate and Calculation Complexity †

**DOI:** 10.3390/s21072306

**Published:** 2021-03-25

**Authors:** Jiun-Yu Sung, Jin-Kai Chen, Shien-Kuei Liaw, Hiroki Kishikawa

**Affiliations:** 1 Department of Electronic and Computer Engineering and Graduate Institute of Electro-Optical Engineering, National Taiwan University of Science and Technology, Taipei 10607, Taiwan; m10719007@mail.ntust.edu.tw (J.-K.C.); skliaw@mail.ntust.edu.tw (S.-K.L.); 2 Department of Optical Science, Tokushima University, Tokushima 770-8506, Japan; kishikawa.hiroki@tokushima-u.ac.jp

**Keywords:** optical sensing, fiber Bragg grating (FBG), peak detection, fitting, regression, filtering

## Abstract

Fiber Bragg gratings (FBGs) are widely applied in optical sensing systems due to their advantages including being simple to use, high sensitivity, and having great potential for integration into optical communication systems. A common method used for FBG sensing systems is wavelength interrogation. The performance of interrogation based sensing systems is significantly determined by the accuracy of the wavelength peak detection processing. Direct maximum value readout (DMVR) is the simplest peak detection method. However, the detection accuracy of DMVR is sensitive to noise and the sampling resolution. Many modified peak detection methods, such as filtering and curve fitting schemes, have been studied in recent decades. Though these methods are less sensitive to noise and have better sensing accuracy at lower sampling resolutions, they also confer increased processing complexity. As massive sensors may be deployed for applications such as the Internet of things (IoT) and artificial intelligence (AI), lower levels of processing complexity are required. In this paper, an efficient scheme applying a three-point peak detection estimator is proposed and studied, which shows a performance that is close to the curve fitting methods along with reduced complexity. A proof-of-concept experiment for temperature sensing is performed. 34% accuracy improvement compared to the DMVR is demonstrated.

## 1. Introduction

Artificial intelligence (AI) is considered as a key element for the on-going industrial revolution and has rapidly developed in recent years. Through the implementation of AI, a completely automatic and convenient world is envisioned; thus, enabling all work to be efficiently and autonomously performed to its maximum capability. Behind this great vision, sensing technologies are also being actively developed in order to perceive the world and respond as essential elements in AI algorithms. For the potential massive deployment of sensors and applications requiring real-time operation with reduced power [1], sensing technology features with fast responses and low-complexity are required.

Fiber Bragg gratings (FBGs) are optical components which feature high levels of sensitivity and can be easily integrated into optical fiber communication systems. Therefore, FBGs have been extensively applied in optical sensing systems. Wavelength interrogation is a common scheme that is used for FBG sensing systems. FBG spectra (peak wavelength) typically change upon variations in specific physical parameters, such as temperature and strain [2]. In an interrogation sensing system, the FBG spectra are continuously monitored. By detecting FBG wavelength shifts, the values of corresponding physical parameters can be estimated.

The most direct option to detect the FBG wavelengths is to monitor the overall system spectrum through an optical spectrum analyzer (OSA) [3]. However, for most commercial OSAs, the wavelength resolution is >0.01 nm. This limits the readout resolution of the physical parameters. Additionally, the scanning speed of OSAs is typically slow, which is disadvantageous for real-time applications. Various different architectures have been proposed to improve the resolution and speed of the interrogation systems by estimating the wavelength shifts through different measurable parameters, such as power [4,5], frequency [6,7], and time [8,9,10]. These parameters can be quickly and accurately measured with mature electronic devices; hence, improving the sensing performance.

There are various peak detection methods which can decide the peak FBG coordinates (wavelengths) from their equivalent spectra (estimated from spectrum, power, frequency, or time, as mentioned above) [11]. Among the various options, direct maximum value readout (DMVR) is the simplest. However, the sensing resolution of DMVR is limited by the circuit sampling rate, which denotes the minimum distinguishable difference between two potential coordinates of a peak. Even if the architectures shown in [4,5,6,7,8,9,10] can be applied, the high-speed electronics may incur an extra cost. Moreover, for practical application scenarios, additional noise sources, such as thermal or shot noise, could further distort the spectrum shapes. While the signal-to-noise ratio (SNR) is low, the spectrum shapes will fluctuate significantly. This causes an extra error when processing peak detection.

Filtering [12,13], correlation [14] and curve fitting [15,16,17] methods have been proposed to reduce the impact of noise. Filtering has shown great capability to reduce peak uncertainty from noise induced intensity fluctuations. However, similar to the DMVR method, the resolution is limited by the circuits’ sampling rate. Other than being insensitive to noise, curve fitting methods can further estimate the peak coordinates between integer sampling points; hence, a finer resolution can be detected with slower electronic devices. Nevertheless, these great advantages of curve fitting result in a higher computational complexity. This increases the power consumption and system cost, hindering the development of intelligent systems from which massive sensors may be deployed. In [16,17], simplified curve fitting schemes were proposed, which indeed, relieve the computational complexity. However, these schemes can be sensitive to noise and may not be suitable for low SNR conditions. As indicated in [11], digital signal processing (DSP) based interpolation/re-sampling techniques have also been considered. However, these approaches, again, increase the computational complexity and cost.

In this paper, a cost-effective and computationally-efficient peak detection scheme is proposed, employing both filtering and dynamic local peak estimation techniques. This scheme is less sensitive to noise and offers finer peak detection resolution, with fewer crucial requirements for high-performance electronic devices. At the same time, with the simplified processing, the calculation complexity is close to the DMVR method. This reduces both the capital expenditure (Capex) and the operational expenditure (Opex) of the systems and can be advantageous for on-going/future intelligent applications. A proof-of-concept temperature sensing experiment was performed. An improvement in accuracy of 34.4% (0.21 °C) was demonstrated. Parts of the paper have been published in The 25th OptoElectronics and Communications Conference (OECC 2020) [10]. In this paper, further analyses and detailed illustrations of the works are provided.

## 2. Theory and Principles

**Figure 1:**
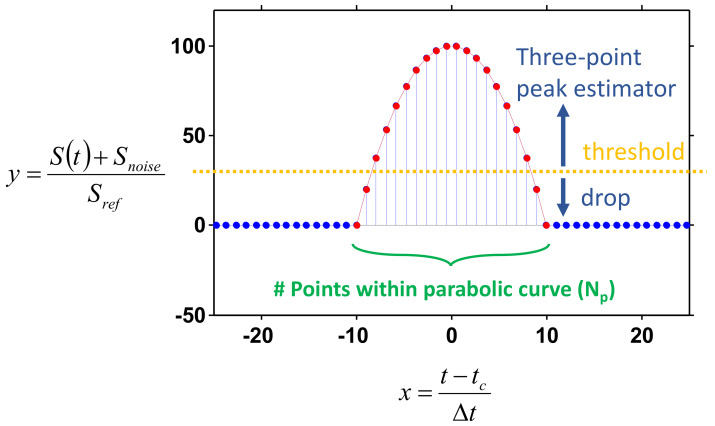
Logical curve for analyses and illustrations. S(t) is the signal time trace.

One FBG is considered for the illustration. The spectrum of this FBG is interrogated before signal processing. Different interrogation architectures can be flexibly applied based on practical considerations such as signal power, noise/distortion, and cost. According to the interrogation architectures, the detected spectrum curve is interpreted by specific physical units. In conventional OSA based detection schemes [3], the *x*-axis of the curve is the wavelength and the *y*-axis of the curve is intensity; in a wavelength-swept-laser (WSL) based scheme [8,9,10], the *x*-axis is time and the *y*-axis is the amplitude of electronic signals. Different physical units can be equivalently transformed based on the practical system setup/parameters. As the performances of different peak detection schemes are mainly affected by the curve’s shape rather than its unit, a logical curve with unitless *x* and *y* coordinates are considered in the below illustration. Figure 1 shows an ideal curve which emulates the detected spectrum of an FBG. For the *x*-axis of the ideal curve, the peak center is offset to the origin, and the physical parameters are normalized with specific scalers. Using the WSL based architecture as an example (labeled in coordinates of Figure 1), the time trace of the FBG reflection response is offset by *t_c_*, resulting in the curve peak being located at the time origin and the time coordinate values are divided by *Δt*. The choice of *Δt* can be arbitrary, based on practical signal trace and the signal processing setup. For example, if the FBG reflection curve is located between time −2~2 s, *Δt* can be set as 4 (2−(−2)) seconds to limit the curve within *x* ∈ [−0.5, 0.5] (assume *t_c_* = 0). All the *x* and *y* coordinates can be equivalently transformed to specific physical parameters, such as time or amplitude, by inverse offset and scale operations. Hence, this normalization process causes no practical influences on peak detection.

**Figure 2:**
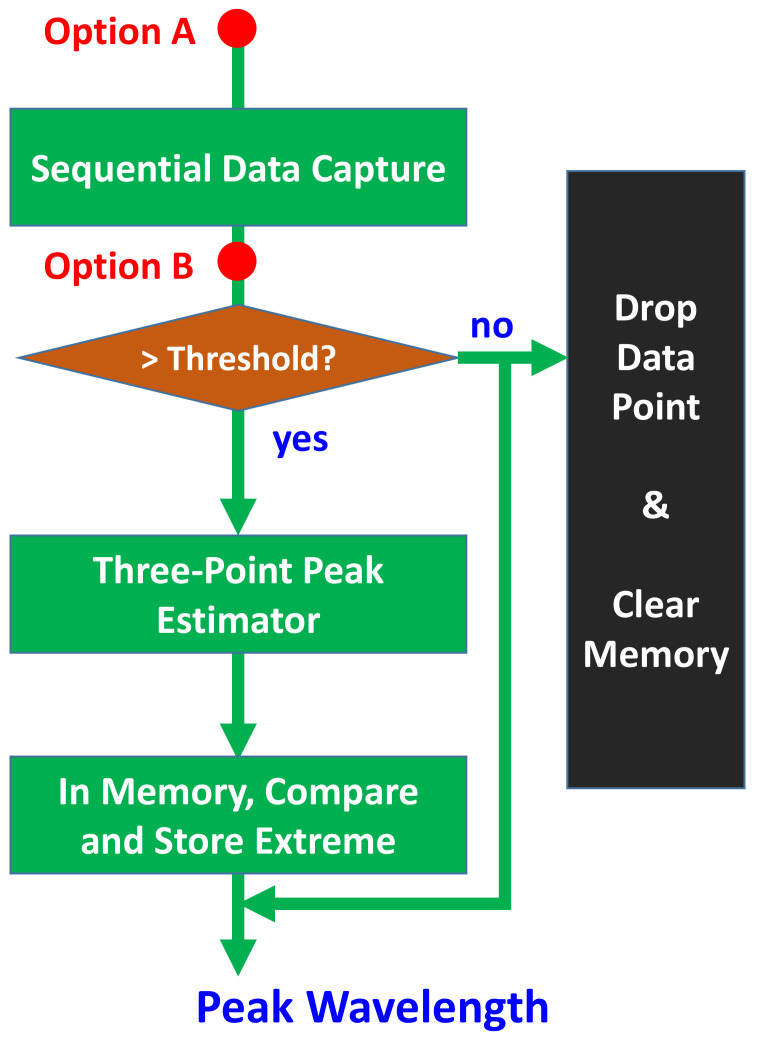
Processing flow of the proposed scheme.

The detected spectrum curve is synchronized in the x-coordinate in the central processor. Then, peak detection is performed, as shown in Figure 2. The spectrum response curve is sampled and captured sequentially. The sampling rate must be sufficiently high to ensure that there is a sufficient number of data points within the region of the FBG peak (this will be discussed below). The intensity of each data point is compared with a pre-set threshold level. All data points with an intensity below this threshold are dropped directly. The three-point peak estimator is triggered once data points with an intensity higher than the threshold level are detected. The selection of data points for different types of processing is briefly shown in Figure 1.

For the three-point peak estimator, it is assumed that the curve region near the peak can be well approximated by a second order polynomial function such as Equation (1):

(1) $$y\left(x\right)=Ax^{2}+Bx+C,$$

where *A*, *B*, and *C* are specific constants best fitting the data points and the quadratic (quad) curve. Two points should be noted. First, a second order polynomial approximation is directly applied without considering the theoretical shape of the FBG spectrum, as was done in [11,12,13,14,15,16,17,18]. Hence, the proposed scheme can work under more general conditions. In the demonstration experiment, it is shown that the presumption of a Gaussian spectrum may not be applicable for general conditions. Second, without knowledge of the theoretical spectrum shape, a second order polynomial approximation can cause an additional estimation error. This error can be reduced by increasing the sampling rate of the data points or including higher-order polynomial terms for processing. Higher-order polynomial terms can be directly added with the same principles illustrated below.

After the sampling process, we have x=n⋅Δx. Then, Equation (1) is expressed in its discrete form as:

(2) $$y\left[n\right]=an^{2}+bn+c,$$

where Δx is the sampling period, n is the index of the sampling points, and *a*, *b*, *c* are constants calculated between *A*, *B*, *C* and Δx. For any three consecutive points, the discrete curve can be re-formulated as Equation (3):

(3) $$\left(\begin{array}{c} y\left[-1\right]\\ y\left[0\right]\\ y\left[1\right] \end{array}\right)=\left(\begin{array}{ccc} 1 & -1 & 1\\ 0 & 0 & 1\\ 1 & 1 & 1 \end{array}\right)\left(\begin{array}{c} a\\ b\\ c \end{array}\right).$$

It is assumed that any three consecutive points locally form a quadratic curve (whether the local spectrum curve can be well approximated by a quadratic curve or not). Hence, during the sequential capturing process, the data point under concern is labeled as *n* = 0, and the data points proceeding and succeeding it are, respectively, *n* = −1 and *n* = 1. This scheme allows real-time processing to sync with the capturing processing, even if all data points of the complete spectrum have not been fully captured. Though the data points away from the actual curve peak cannot be accurately approximated by a quadratic curve, their calculation results will typically be dropped during the succeeding comparison processing (as illustrated below). Hence they will not influence the detection results. By a simple matrix operation, Equation (4) can be deduced from Equation (3) as:

(4) $$\left(\begin{array}{c} a\\ b\\ c \end{array}\right)=\left(\begin{array}{ccc} 0.5 & -1 & 0.5\\ -0.5 & 0 & 0.5\\ 0 & 1 & 0 \end{array}\right)\left(\begin{array}{c} y\left[-1\right]\\ y\left[0\right]\\ y\left[1\right] \end{array}\right).$$

According to Equation (4), coefficients *a*, *b*, and *c* of the local quadratic curve are determined by three consecutive data points. Then, the extreme value of the local quadratic curve is determined as (“extreme” rather than “maximum” values are denoted here, as a parabolic curve may have either upward or downward polarity):

(5) $$Extreme\left\{y\left[n\right]\right\}=c-\frac{b^{2}}{4a},\;n=-\frac{b}{2a},$$

From Equation (5), it can be seen that n can be a fractional number. Hence, even if the actual peak is between the sampling points, it can be well estimated. Then, a finer sensing resolution, lower than the limitation of the sampling rate, can be performed.

In the three-point peak estimator, the extreme values of all local quadratic curves are sequentially calculated according to Equations (4) and (5). There are possibilities that the extreme values of the local quadratic curves away from the actual peak are higher than the actual peak for abrupt curve shapes. However, under these conditions, the peak location calculated from Equation (5) is typically at *n* > 1 or *n* < −1. As the main purposes of the parabolic approximation are to improve the sensing resolution, it is reasonable to assume that if the *n* > 1 or *n* < −1 peak locations are parts of the actual peak, it will be calculated from points closer to the peak again (because the fractional portion of n is mainly concerned). Hence, we can neglect the *n* > 1 or *n* < −1 results to avoid the fake peak locations. In the processing unit, a dedicated processing register is initially set with its minimum allowed value. The calculated extreme values with $\left|n\right|<1$ are compared with the value stored in the processing register. The bigger extreme value will be continuously held and updated by the processing register. The three-point peak estimator and comparison processes terminate, while the threshold condition in the preceding stage is violated. As the FBG spectrum curve is continuous, the values of the data points will decrease after the peak location. Once the values of the data points return to below the threshold level, the whole peak region passes. Then, according to the extreme value sampling index stored in the register, the peak location is estimated. For systems with multiple sensing units (multiple FBGs with different spectrum peaks), the same process can be continuously performed to detect multiple spectrum peaks. The whole process is repeatedly performed to dynamically update the sensing information.

The operational processes of the proposed method are similar to the DMVR method. With only a slight increase in processing cost, the sensing accuracy can be improved with lower speed sampling circuits. Hence, this can be a cost-effective alternative to real-time and low-complexity sensing applications. One key issue of this method is that it is sensitive to noise, as is the case with the conventional DMVR method. To enable this method to work at low SNR conditions, further low-pass filters preceding (option A of Figure 2) or inside (option B of Figure 2) the processing chain can be applied to reduce the noise induced signal fluctuations. Digital based filtering schemes have been studied in [12,13].

It should be noted that this proposed method is different to conventional curve fitting methods. Conventional curve fitting methods take the wide spectrum region for regression operations. Through the statistical information of large numbers, curve fitting methods are typically insensitive to noise and can obtain great sensing accuracy. However, according to the simulation results of [11,16], >20 data points are typically required to achieve a fitting accuracy close to the best capable accuracy level. The increasing number of calculation data points results in higher system complexity and energy consumption. On the other hand, conventional curve fitting methods also take extra time to capture the whole spectrum data, and additional windowing operations are necessary to select the spectrum portions to be fitted [16,18]. It can also be noticed that similar N-point peak estimator processing is studied in [16,17], merely as a simplified curve fitting alternative. However, in both [16,17], the processing simplification is based on high SNR presumptions (SNR > 20 dB and SNR > 40 dB are considered, respectively, in [16,17]). This causes the performance to be sensitive to noise and losses the advantages of conventional curve fitting methods. Additionally, windowing operations and knowledge of multiple spectrum data points are also required, which increase the memory and time expense. It is also worth mentioning that the windowing method, based on the maximum value selection used in [16], neglects the potential side lobe effects from the FBG spectra, as illustrated in [18]. Through our proposed scheme, within each continuously connected data point set above the threshold level, it is guaranteed that only one peak will be estimated. Hence, by appropriately selecting the threshold level, the potential FBG side lobes will not be incorrectly judged as another FBG peak.

## 3. Performance Analyses

The performance at different conditions for our proposed scheme was further analyzed. The following parabolic curve was emulated as the FBG noiseless amplitude response.

(6) $$\left\{\begin{array}{cc} y=-x^{2}+100,\; & y>\mathrm{noise}\;\mathrm{level}\\ y=0, & Otherwise \end{array}\right..$$

**Figure 3:**
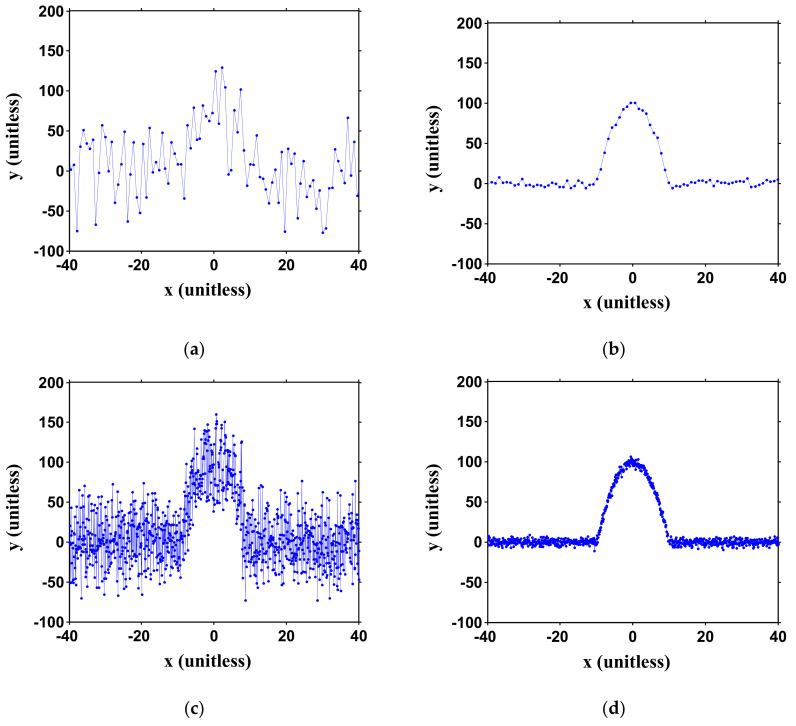
Emulated captured curves under different conditions. (The ideal curve above the noise level is set as *y* = −*x*^2^ + 100). Left column (**a**,**c**): signal-to-noise ratio (SNR) = 10 dB; right column (**b**,**d**): SNR = 30 dB; top row (**a**,**b**): *N_p_* = 20; bottom row (**c**,**d**): *N_p_* = 200. *N_p_*: number of points within the parabolic curve. Curves are unitless as illustrated in Figure 1.

It should be noted that the physical units have been normalized, as explained above. Gaussian noise is directly added to Equation (6) with its intensity meeting the pre-set SNR conditions. Different numbers of sampling points (*N_p_*) within the parabolic region are also considered. The relation between *N_p_* and the emulation is illustrated in Figure 1. Conditions of SNR = 10 and 30 dB and *N_p_* = 20~200 are studied. It should be emphasized that *N_p_* is only relevant to the signal sampling rate. In our three-point peak estimator, only the consecutive three points are processed for each calculation. The generated curves at different conditions are shown in Figure 3. It can be seen that for SNR = 10 dB, the parabolic curve fluctuates greatly. For SNR = 30 dB, the parabolic curve can be clearly identified but observable curve fluctuations still exist. An interesting finding is that, for *N_p_* = 200 (high sampling rate), the ambiguity to directly detect the peak through DMVR is even bigger than the case of *N_p_* = 20 (low sampling rate). Though the high sampling rate can obtain a finer sensing readout resolution, the potential noise-induced peak detection error can dominate the potential advantages. The accuracy (resolution) improvement that is obtainable from the proposed three-point peak estimator, will be greatly reduced by this phenomenon. Comparing this with Figure 3b,d, it is possible to reduce the peak ambiguity with a lower sampling rate. However, this will reduce the sensing readout resolution.

**Figure 4:**
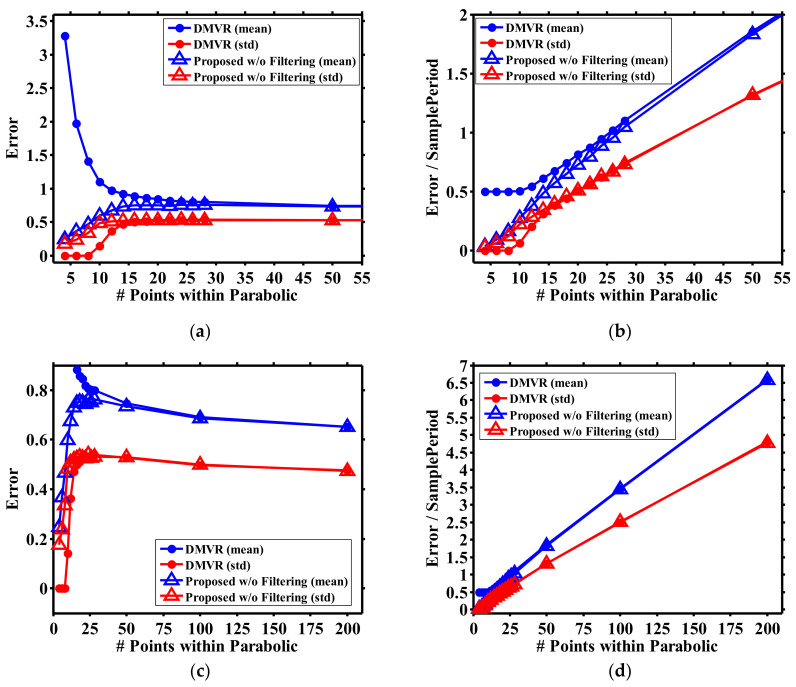
Peak detection performance at SNR = 30 dB. (**a**–**d**) are, respectively, plotted with the same data, but enlarged at different regions of the coordinate system.

Figure 4 shows the peak detection error for the DMVR and our proposed scheme at SNR = 30 dB. Comparing Figure 4a,c, it is observed that the low sampling rate results in reduced detection error for our proposed scheme, as explained for Figure 3b,d. However, DMVR performs less well at low sampling rates, which instinctively contradicts the discussion for Figure 3b. This can be explained by Figure 4b,d, which normalized the error in the unit of the sample period. With this coordinate transformation, the detection errors increase with a higher sampling rate for both schemes. From this direct observation, it is emphasized that a low sampling rate results in reduced peak ambiguity, but this does not always lead to a low detection error. At high sampling rates, though there may be more ambiguous peaks, these peaks may locate within a limited region. Though this region may occupy multiple sample periods, it can be sufficiently small compared to the final readout error. Since the detection error is not dominated by the peak ambiguity, the error at high sampling rate can be lower than expected. From Figure 4a,b, it can also be seen that the proposed scheme offers significant accuracy improvements at low sampling rates. This improvement degrades with the increases in peak ambiguity. Comparing Figure 4c,d, though more ambiguity is observed at higher sampling rates, the detection error still slowly decreases with higher sampling rates. Finally, as a reference to the next discussion, the detection error is approximately 0.25 at *N_p_* = 4 (for the best performance), which is about 3~10 times (respectively, for the worst and best conditions for the following discussion) higher before the following filtering operation is performed.

**Figure 5:**
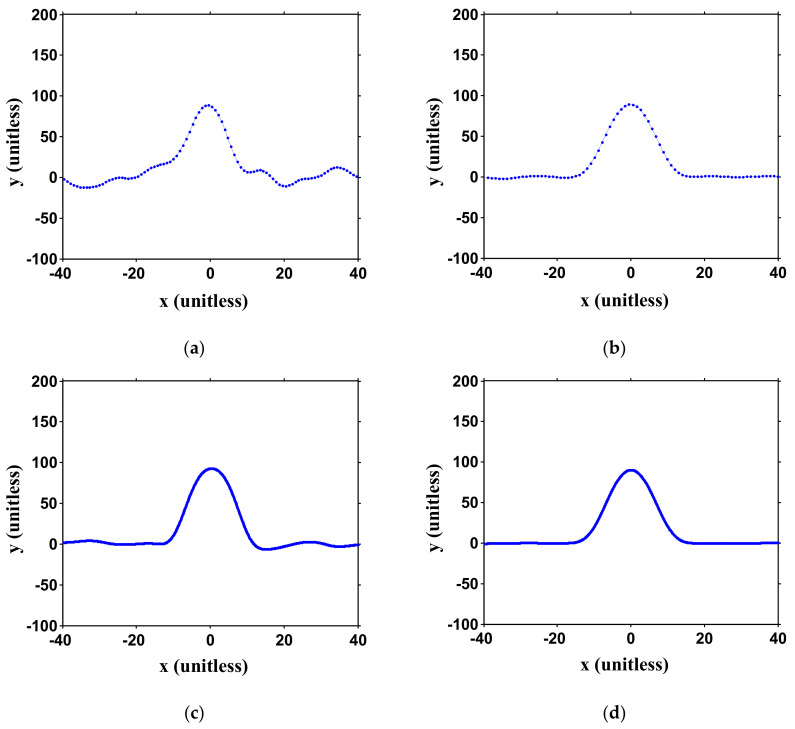
Emulated captured curves under different conditions after filter. The ideal curve above the noise level is set as *y* = −*x*^2^ + 100. Left column (**a**,**c**): SNR = 10 dB; right column (**b**,**d**): SNR = 30 dB; top row (**a**,**b**): *N_p_* = 20; bottom row (**c**,**d**): *N_p_* = 200. *N_p_* : number of points within the parabolic curve. Curves are unitless, as illustrated in Figure 1.

A digital filter is added as option B of Figure 2 to reduce the noise. The signal curves after the filter are shown in Figure 5. It can be observed that the peak ambiguity for all SNRs and sampling conditions is greatly reduced. The three-point peak estimator can then be applied to the signal to obtain a finer resolution within the sampling period.

**Figure 6:**
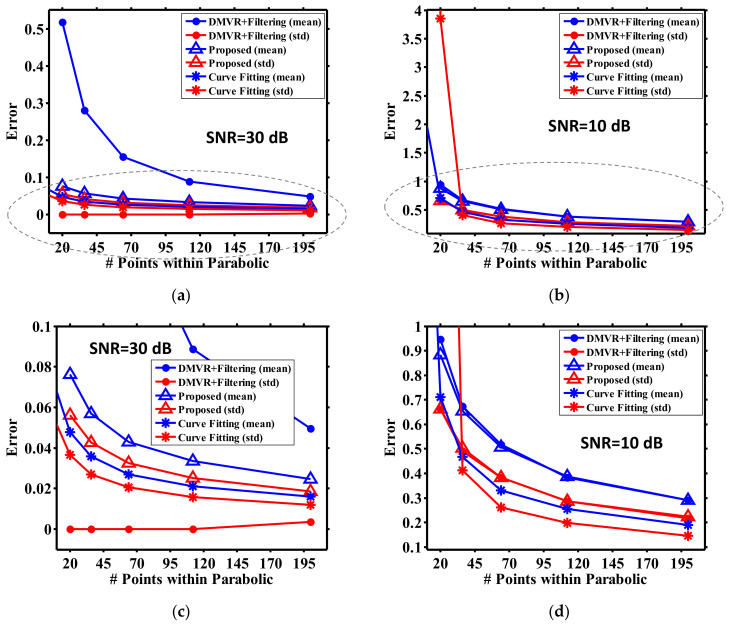
Peak detection performance for different schemes. (**a**,**c**) for SNR = 30 dB; (**b**,**d**) for SNR= 10 dB. (**c**,**d**) are regionally enlarged from (**a**,**b**) (the regions circled with dash lines).

The performance improvements of different peak detection schemes were studied, as shown in Figure 6. Here, the curve fitting method (without filtering processing) is also included as a reference, denoting the potential best available sensing accuracy. The error metric used in Figure 6 is the absolute location deviation between the estimation results and the theoretical values (i.e., zero according to Equation (6)). As the error deviation of DMVR can cross a wide range, Figure 6c,d is regionally enlarged from Figure 6a,b to more clearly show the results of different applied schemes. The simulation was performed 5000 times. Each time the noise pattern was updated with the same intensity level. The average and standard deviation of the estimation errors were both calculated.

For all conditions, it can be seen that that the errors of all peak detection methods converge at a high sampling rate. As there are error floors from inherent system factors, the accuracy obtainable from all methods is inevitably limited. Hence, at high sampling rates, the accuracy of DMVR can already be high, and the advantages of the three-point peak estimator and curve fitting become less significant.

As shown in Figure 6a, for high SNR conditions, the three-point peak estimator can achieve approximately an 80% improvement compared to the DMVR methods at a lower sampling rate (*N_p_* = 20), and the DMVR’s performance is close to the other two methods, while *N_p_* > 200. This reveals that the proposed method can relieve the requirements of the sampling circuits by more than 10 times under a guaranteed accuracy.

From Figure 6c, it can be seen that curve fitting still has the best performance. We believe this shows the curve fitting method is more insensitive to noise induced signal fluctuations. The filter may be further optimized to obtain an error that is close to the curve fitting methods. However, customization for both the filter response and curve fitting require pre-knowledge of the practical shape of the curve; hence, this makes the system less flexible. On the other hand, though the DMVR method has a worse average error performance, its standard deviations (over the 5000 runs) are low for all sampling conditions. This is reasonable because it is mainly limited by the sampling error and influenced less by the processing error.

Figure 6b shows that curve fitting becomes less robust at the low SNR condition, while the sampling rate is low. This may come from the fact that the number of data is insufficient to statistically reduce the impacts of noise. Hence, the curve fitting processes are greatly dominated by the behaviors of noise. This results in a random peak estimation and higher errors. As we mentioned above, more sampling points are then further required for curve fitting to guarantee the sensing accuracy, and the system complexity will increase.

From Figure 6d, it can be seen that, at low SNR conditions, the proposed three-point peak estimator and the DMVR method have similar performances. Typically, the three-point peak estimator should estimate peak locations within two consecutive samples and obtain a better level of accuracy. However, it should be noted that the error in Figure 6d is almost higher than that in Figure 6c by an order. This reveals that the error is inherently dominated by the filtering processes, which causes an error almost 10 times higher than the improvement offered by the three-point peak estimator. This greatly reduces the function of the three-point peak estimator. Moreover, while the sampling rate is high, curve fitting still performs better than the other two methods. We believe this is dominated by the same reasons as explained for Figure 6c.

**Figure 7:**
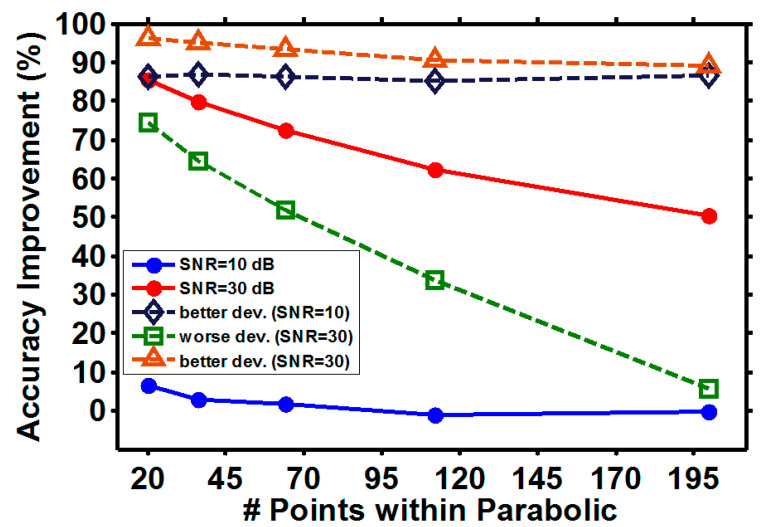
Accuracy improvement under different SNR conditions. Dash line: the potential variation induced from performance fluctuation of different schemes.

In particular, the accuracy improvement of our proposed scheme from the DMVR method is discussed, as shown in Figure 7. At high SNR (SNR = 30 dB) conditions, the average relative accuracy improvement reduces, as the sampling rate is high. This corresponds to the discussion for Figure 6a. However, even if the number of data points within the parabolic curve is 200, a ~50% improvement can still be anticipated. Considering the standard deviation of the errors, the green-square and brown-triangular curves show the potential improvement variations from the average results. It can be seen that a more than 90% potential improvement can be expected even at high sampling rates, which instinctively contradicts the discussion of Figure 6a. This comes from the fact that the average and standard deviation errors converge towards specific floor levels at high sampling rates. As shown in Figure 6, the floor levels are higher than zero for all conditions. Hence, at high sampling rates, while the performances between the two schemes converge, the random accuracy fluctuations cause that the DMVR method occasionally performs worse than the proposed scheme. Consequently, a high accuracy improvement can still be expected at a high sampling rate. However, this is just a random behavior and has no practical information. On the other hand, the performance improvement of the proposed scheme can also degrade even more quickly than the average improvement. It can be seen that, while the number of data points within the parabolic curve is 200, the improvement can be negligible with the effects of random accuracy fluctuations. At the low SNR (SNR = 10 dB) condition, the average accuracy improvement from our proposed method is small. This corresponds to the discussion of Figure 6d. Similar to the high SNR condition, higher accuracy improvements can be expected once the random accuracy fluctuations are considered, as shown in the dark-blue diamond dash line. The difference between the levels of improvement is determined by the relative levels between the converged average and standard deviation error floors. The reduced accuracy improvement line from random fluctuations is not plotted for the low SNR condition. It can be seen that the average accuracy improvement is already small. With the considerations of the random fluctuations, DMVR can sometimes offer better accuracy than the proposed method. This will result in a negative accuracy improvement. However, as the results fall behind random behaviors, as discussed above, they do not provide any meaningful information.

Our summarized conclusions are presented here. The proposed three-point peak detection estimator can improve the sensing accuracy compared to the DMVR method. Improvements as high as 80% can be anticipated at low sampling rates and high SNR conditions. Though the improvement degrades at high sampling rate and low SNR conditions, it at least offers a similar performance to the DMVR method. Moreover, while the improvement becomes less significant, the performance is also closer to the curve fitting method. Curve fitting performs worse at low SNR and low sampling conditions. Hence, the requirements of the sampling circuits and the calculation complexity are even higher than those that are sufficient for the high SNR conditions. Once the standard deviation is included in the consideration of the potential random accuracy fluctuations, the best accuracy improvement can always, in a probabilistic way, be high, depending on the relative levels between the average and standard deviation error floors. The improvement of the proposed method can also be further degraded by the random fluctuations. While the number of data points within the parabolic curve is >200, the worse accuracy improvement can become negligible even at high SNR conditions. As a reference, the performance among different schemes is roughly compared in Table 1. In the comparison, it is presumed that all parameters, other than the one under consideration, are adjusted to the best possible levels. However, it must be noted, that from the above discussion, the performances of different schemes are co-determined by multiple operational parameters. Hence, Table 1 can over-estimate the practical performance of the schemes.

## 4. Experiment

**Figure 8:**
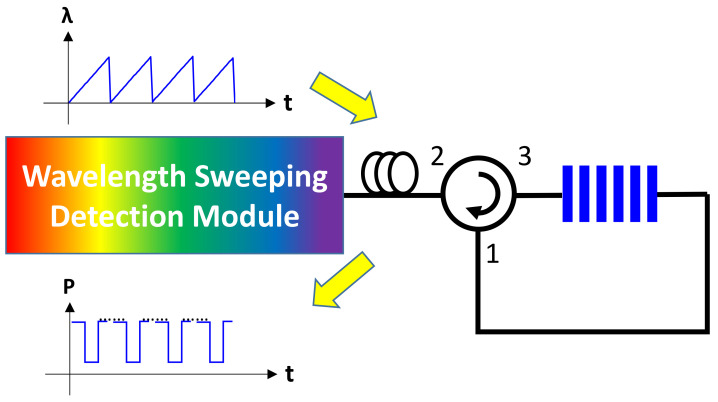
Experiment setup.

A proof-of-concept experiment based on the same architecture reported in [9,10] was performed, as shown in Figure 8. The wavelength sweeping detection module is composed of a wavelength swept laser (WSL), a reference FBG, and a corresponding receiver module. The receiver module comprises an optical power meter connected to the oscilloscope. The WSL linearly sweeps the transmitted optical wavelength over time to the FBG sensor head. The re-circulated loop is performed by an optical circulator (OC). The optical signal passes the FBG through the OC. The optical wavelengths within the FBG reflection band are reflected back into port-3 of the OC and leave the system; other wavelengths pass the FBG, and are re-circulated back into the wavelength sweeping detection module through the OC. The re-circulated optical signal is detected by the receiver module and its time trace is recorded by the oscilloscope with a sampling rate of 25 kS/s. As described above, the advantages of the three-point peak detection cannot be significantly observed; hence, the received signal is down-sampled by a factor of 200, which corresponds to 125 Sample/s. Then, the processes illustrated in Figure 2 are performed offline. In order to test sensing performance for practical physical parameters, the FBG is heated to different temperatures, and the signals under different temperatures are processed. For each temperature, three curves are measured and processed independently to show the potential detection fluctuation under the same temperature.

**Figure 9:**
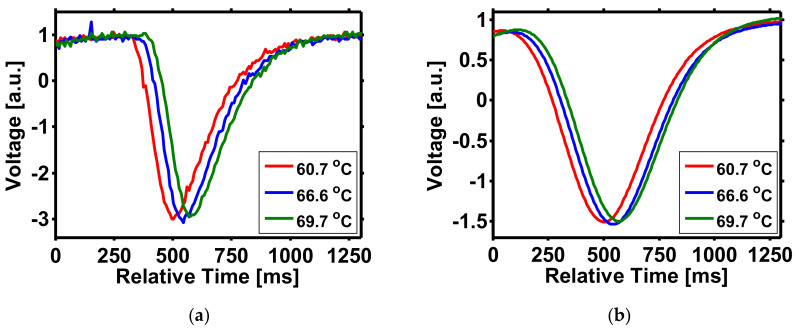
Three of the received curves (**a**) before and (**b**) after the filter.

The time traces of the captured signals for different temperatures are shown in Figure 9. It can be observed that the signals for different temperatures shift in association with the relative detected time. The time shifts can be transformed to equivalent wavelength shift values, as described in [8,9,10]. Due to the impact of noise, the received signal traces fluctuate at an observable level, as shown in Figure 9a. As described in Figure 3, this will degrade the accuracy of the peak detection processes because of the ambiguous peaks. In order to eliminate the noise, a 1.25 Hz digital low-pass filter is applied. The peak ambiguity is greatly eliminated by the filtering process, as shown in Figure 9b. It should be noted that the received curve is not parabolic nor Gaussian as many studies have pre-assumed [11,12,13,14,15,16,17,18]. We believe this special curve shape comes from the slow response of the power detector and can further vary while different receivers are applied. However, this reveals the potential that, in a more general application scenario, the great sensing performance from curve fitting may be degraded to a greater degree than is anticipated because of unexpected factors which change the curve shapes.

## 5. Discussion

**Figure 10:**
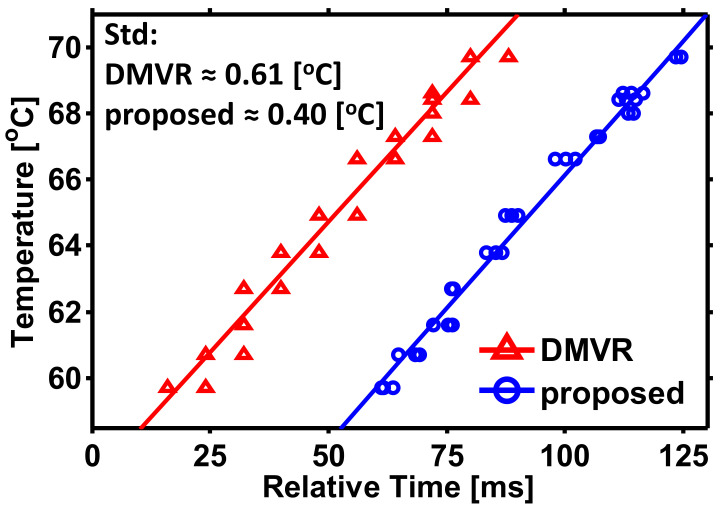
Relation between the temperatures and the relative time shifts. Temperature range: 59.7~69.7 °C.

The sensing performance of the proposed scheme compared to the DMVR method is shown in Figure 10. It can be observed that the temperatures and the relative peak time shifts behave in a linear relation. The parameters of the regression line are shown in Table 2. In order to clearly show two curves in the same picture, the *x*-axis of the curves is intentionally shifted. As only the relative peak time shifts are physically meaningful for the interpretation of temperatures, this will not influence the discussion of the sensing performance. According to the slopes of the curves, both curves show sensitivities of about 0.16 °C/ms. For each value of the peak time shift (i.e., variable *t* of the curve), the calculated temperature (*T*) on the regression curves are considered as the ideal value. The ideal value is compared with the measured result. The standard deviations for both processing schemes are, respectively, 0.6133 °C and 0.4021 °C. Through the proposed method, about 34% relative accuracy improvement is observed. The correlation coefficient of the proposed scheme is also higher than the results calculated for the DMVR method. This comes from the grid-like behavior of the DMVR data points. As mentioned above, the readout resolution of DMVR is limited by the sampling rate. Even if the peak is appropriately detected by DMVR, the peak coordinates only locate at the grids of the integer sampling period. This causes the data points in Figure 10 to jump discretely for DMVR and reduces the final correlation coefficient. It can be seen that through the proposed scheme, the peak coordinates are not limited to the grids of integer sampling period, and the correlation coefficient increases.

## 6. Conclusions

A simple but efficient peak detection scheme is proposed and studied. Through the proposed scheme, the data are processed immediately once they are captured. No specific windowing operation is required. All processing is performed with few data points. This speeds up the sensing processing for real-time applications. A reduction in the number of processed data points also reduces the hardware buffer size. The three-point peak estimator can further improve the resolution/accuracy of the peak readouts, which reduces the high-speed requirements of the electronic devices. This further reduces the potential system cost. Performance analyses among the proposed scheme, DMVR, and curve fitting were performed. It is estimated that the proposed scheme can obtain a similar accuracy level with the sampling rate about an order less than the DMVR method. It is estimated that more than 20 samples are required for the curve fitting methods to obtain a stable detection accuracy at low SNR conditions. Though the performance of the proposed scheme is slightly degraded compared to the curve fitting methods, it uses much fewer computational and hardware resources. A temperature sensor was built as a proof-of-concept experiment. Through the proposed processing scheme, a ~34% accuracy improvement compared to the DMVR is demonstrated.

## Figures and Tables

**Table 1:** Comparison of different schemes

Scheme	Accuracy	Sampling Ratio	Complexity	Noise Robustness
DMVR	Low	High	Low	Low
Curve fitting	High	High	High	High
Proposed	Between	Low	Between (Close to DMVR)	High

**Table 2:** Parameters of the regression curve T=A⋅t+B.

Scheme	A (°C/ms)	B (°C)	σ (°C)	Correlation Coefficient
DMVR	0.1572	56.8538	0.6133	0.9827
proposed	0.1616	49.9930	0.4021	0.9926

## Data Availability

The data presented in this study are available on request from the corresponding author. The data are not publicly available due to privacy restrictions.
